# Effects of Virtual Reality Training on Upper Limb Function and Balance in Stroke Patients: Systematic Review and Meta-Meta-Analysis

**DOI:** 10.2196/31051

**Published:** 2021-10-12

**Authors:** Jinlong Wu, Aihua Zeng, Ziyan Chen, Ye Wei, Kunlun Huang, Jiafeng Chen, Zhanbing Ren

**Affiliations:** 1 Department of Physical Education Shenzhen University Shenzhen China; 2 Shanwei Polytech Shanwei China; 3 Nanshan District Culture, Radio, Television, Tourism and Sports Bureau Shenzhen China

**Keywords:** virtual reality, stroke, motor function, review, meta-meta-analysis, mental health, motor skills, rehabilitation, digital intervention, health care, stroke patients

## Abstract

**Background:**

Virtual reality (VR) training is a promising intervention strategy that has been utilized in health care fields like stroke rehabilitation and psychotherapy. Current studies suggest that VR training is effective in improving the locomotor ability of stroke patients.

**Objective:**

This is the first meta-meta-analysis of the effects of VR on motor function in stroke patients. This study aimed to systematically summarize and quantify the present meta-analyses results of VR training and produce high-quality meta-meta-analysis results to obtain a more accurate prediction.

**Methods:**

We searched 4 online databases (Web of Science, Scopus, PubMed, and Chinese National Knowledge Infrastructure) for meta-analysis studies. After accounting for overlap, 10 studies (accounting for almost 550 stroke patients) were obtained. Based on the meta-meta-analysis of these patients, this study quantified the impact of VR training on stroke patients’ motor performance, mainly including upper limb function, balance, and walking ability. We combined the effects under the random effect model and pooled the estimates as standardized mean differences (SMD).

**Results:**

The results of the meta-meta-analysis showed that VR training effectively improves upper limb function (SMD 4.606, 95% CI 2.733-6.479, *P*<.05) and balance (SMD 2.101, 95% CI 0.202-4.000, *P*<.05) of stroke patients. However, the results showed considerable heterogeneity and thus, may need to be treated with caution. Due to the limited research, a meta-meta-analysis of walking ability was not performed.

**Conclusions:**

These findings represent a comprehensive body of high-quality evidence that VR training is more effective at improving upper limb function and balance of stroke patients.

## Introduction

Stroke is the most common cause of chronic physical disabilities, such as dyskinesia. Most stroke patients have dyskinesia, which causes different degrees of impediment to upper limb function, walking ability, and balance. Because of limited exercise ability, these patients cannot participate in daily activities; thus, their quality of life is reduced [1,2]. Stroke rehabilitation mainly aims to help patients return to society and work [3]. Therefore, it is important to comprehensively understand the severity of stroke; improve treatment methods; reduce the incidence, disability, and mortality of stroke; and find safe and effective treatment for stroke patients.

In the past decade, virtual reality (VR), as a means of neurological rehabilitation for stroke, has gradually become popular in the field of rehabilitation because of the continuous improvement in virtual systems and the substantial reduction in cost of virtual equipment [4]. VR technology is a system that can simulate the environment, scene, and activity in real time and allow users to interact through multiple sensory modalities. The system can be combined with a treadmill, bionic gloves, or robots to provide better feedback for users [5]. Moreover, in the virtual rehabilitation scene created by VR technology, the content, duration, and intensity of the exercise can be manipulated, and even timely feedback can be obtained so as to provide users sufficient and personalized exercise [6]. The following VR-related technologies are widely used in the treatment of stroke patients: an innovative exoskeleton, VR telerehabilitation system, IREX immersion VR systems, Xbox Kinect, keyboard with VR, VR combined with gloves, Nintendo Wii, and virtual surfaces [6]. Compared with traditional rehabilitation, the main advantage of VR training is that stroke patients can think of it as an exciting game rather than as treatment; VR training can help users focus their attention completely on the task, thus improving motivation and treatment compliance, which can be of great benefit in recovering from poststroke trauma [7].

In the last 20 years, a large number of studies have confirmed that VR training has certain advantages in improving the condition of people with dyskinesia [8,9]. Among these studies, nearly 300 experimental and more than 60 meta-analysis studies of stroke patients have been published in international journals. However, between the different meta-analysis studies, there is an inconsistency in the effect size of VR training to improve the different exercise abilities of stroke patients. The purpose of this study was to aggregate high-quality evidence from randomized trials and quantify the effects of VR training on the exercise performance of these patients. Meta-meta-analysis is the meta-analysis of meta-analyses and follows the primary outcome research. Moreover, the overlap of primary studies in a meta-analysis is also fully considered in order to clearly illustrate the effectiveness of VR training in improving motor performance. These findings are expected to be the highest quality of evidence to date. Meanwhile, the methodological quality of the included meta-analyses was evaluated to provide valuable information for future research and practice and to help clinical rehabilitation practitioners better understand the potential benefits of VR training.

## Methods

### Search Strategy

Two researchers independently searched for meta-analysis articles published in the following databases: Web of Science, Scopus, PubMed, and China National Knowledge Infrastructure. They used 3 sets of keywords: (1) meta, meta-analysis; (2) stroke, poststroke; (3) virtual reality, virtual game, virtual video, Nintendo Wii, Kinect, Xbox, exergame. The Chinese versions of the aforementioned keywords were retrieved from the Chinese database. The search time frame was from the establishment of the database to November 25, 2020. A complete record of search strings is provided in Multimedia Appendix 1, and the PubMed search strategy is provided as an example. In addition, the reference lists of eligible studies were manually searched to ensure that all meta-analysis studies were included as much as possible**.**

### Study Selection

In this study, 2 researchers independently screened articles by title, abstract, and full text. Before reaching a consensus, differences between the 2 researchers were discussed. If no agreement was reached, a third researcher made the final decision after a group discussion. First, the titles and abstracts were screened, and the following studies were excluded: (1) not a meta-analysis; (2) did not involve related topics, including upper limb function, balance ability, and walking ability (hand function was excluded from upper limb function for research homogeneity); (3) focused on nonstroke patients; (4) used languages other than English or Chinese; or (5) was not published in peer-reviewed journals (ie, excluding conference abstracts, book chapters, and reviews). Next, the full text was screened to exclude meta-analyses with unclear participants or topics. The included meta-analyses had to be randomized controlled trials; other related meta-analyses, such as observational research and longitudinally designed research, were excluded. VR technology can only be used as a general term at present. However, in order to further homogenize the included meta-analyses, it was necessary to define VR technology. The intervention measures of VR technology had to involve building a VR platform. Patients can interact with the electronic screen through motion sensors, accelerometers, gyroscopes, machine gloves, and other devices, and there is no need for them to grasp or manipulate any real objects. In addition to excluding non-VR, computer program–aided meta-analyses, those with unclear statistics were also excluded.

### Data Extraction

A table was designed to extract relevant information. When the 2 researchers had differences during the data extraction process, they discussed and resolved these differences between themselves. If the data were not transparent or lacked relevant information, the author was contacted as much as possible to obtain specific information and improve the quality of this research. If the sample size was not directly stated, the researcher calculated the sample size as accurately as possible from the experimental study. If only the total sample size was reported, these samples were divided into 2 parts to determine the experimental group’s and control group’s approximate values.

If there were multiple measurements for the final indicators, we used the Fugl-Meyer Assessment Upper Extremity Scale (FMA-UE) as the primary measurement for upper limb function, the Berg Balance Scale (BBS) for balance, and Timed Up and Go for walking ability. In addition, in order to obtain more reliable data, the first choice for this study was to extract data comparing the effects of VR training with those of conventional rehabilitation training.

### Quality Assessment

The meta-meta-analysis quality was evaluated by 2 researchers who independently coded using an 11-item multisystem evaluation tool (Assessment of Multiple Systematic Reviews) [10], which proved to be of the right consistency, reliability, and content validity. The scoring criteria were 1 for “yes” and 0 for “not met,” “not applicable,” or “not reported.” The total score of each meta-analysis was the sum of the 11 items, and the quality evaluation was set as low quality (0-4), medium quality (5-8), or high quality (9-11). The final score for each meta-analysis was the average score between the 2 researchers, and the meta-analyses assessed as low quality were excluded. After the included studies were coded, the researchers discussed the coding divergence and used SPSS Version 22 (IBM Corp, Armonk, NY) to assess the intraclass correlation coefficients (ICCs) in order to determine the reliability between the evaluators: 1.00 means complete agreement, and 0.00 means absolute difference [11].

### Correction for Overlap of Basic Research

According to the research results of Munder et al [12] and considering that the overlap of research in a meta-meta-analysis may lead to distortion of the results, this study took the overlap of the main results into account and corrected for the overlap of initial studies. When an experimental study was included in multiple meta-analysis studies, it only contributed to this meta-meta-analysis. This was mainly completed by determining the number of meta-analyses of each significant study. For each meta-meta-analysis, the uniqueness of each included meta-analysis was added to set the adjusted research number: kadj. Finally, a meta-analysis with k_adj_ ≤3 was excluded.

### Data Analysis

The information included in this study and the conclusions on the benefits of VR training in improving stroke patients’ motor performance were from the meta-analyses we rated as medium or high quality. A meta-analysis of the effect size of VR training was conducted under the random effect model of multiple motor abilities. Only 1 effect size was extracted for each meta-analysis. Comprehensive Meta-Analysis (CMA) software 2.0 was used to combine the effect size. CMA is a weighted estimation of effect size based on the method by DerSimonian and Laird. The effect size of each meta-analysis was converted into a standardized mean difference (SMD) to obtain the overall effect size. The effect size was quantified as large (SMD >0.8), medium (SMD 0.5-0.8), or small (SMD 0.2-0.5) [13,14].

The weighted estimation of heterogeneity in all of the meta-analyses was quantified as I^2^ (because heterogeneity is not accidental, but instead is the percentage of total variation among studies), with 25%, 50%, and 75% representing low, medium, and high, respectively. For those using the Q value, the heterogeneity score needed to be re-estimated for cross-review comparison. Larger values indicated higher heterogeneity, and *P*<.05 indicated significant differences in heterogeneity [15]. Because of the small number of included studies, further subgroup analysis and sensitivity analysis were not conducted in this study.

### Publication Bias

Publication bias is defined as “the bias caused by the nature and direction of research that influences the decision whether to publish or otherwise distribute it” [16], which leads to an unknown number of unpublished negative results. Since there were less than 10 meta-analysis articles for each result in this study, no funnel plot was generated, and the Egger test was used to evaluate the publication bias [17].

## Results

### Literature Search

**Figure 1:**
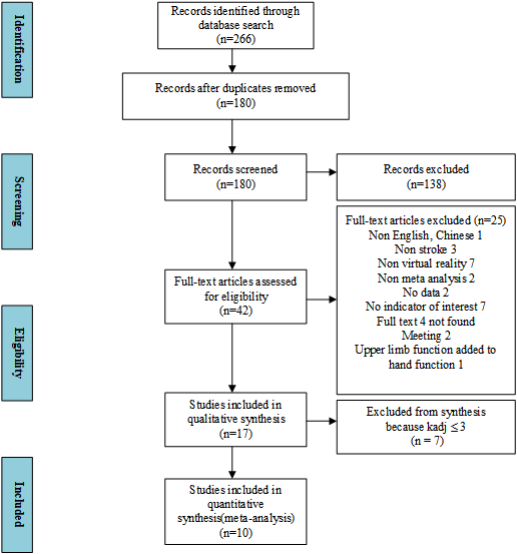
PRISMA (Preferred Reporting Items for Systematic Reviews and Meta-Analyses) flow diagram for studies included in and excluded from the meta-meta-analysis.

A total of 226 records was retrieved from the database, and 180 records remained after removing the duplicate records. A total of 138 records was deleted because of their unqualified titles and abstracts. A more detailed full-text screening was performed with the remaining 42 studies, of which 25 were excluded because they did not meet the established criteria. Therefore, the qualitative research included 17 studies. An additional 7 studies were excluded because k_adj_ was ≤3, and therefore, the final quantitative analysis included a total of 10 meta-analysis studies (Figure 1).

### Research Characteristics

The characteristics of all the included studies are shown in Tables 1-3. A total of 10 studies was obtained: 6 studies evaluated the rehabilitation of upper limb function (Table 1), 4 studies evaluated the rehabilitation of balance (Table 2), and 2 studies evaluated the rehabilitation of walking function (Table 3).

**Table 1:** Characteristics of the included studies for upper limb function.

Author(s), year	Aim	k^a^	Sample size	Measuring instrument	Effect size			I^2^	k_adj_
					Effect size	95% CI (LL^b^ to UL^c^)	*P* value		
Yang et al, 2013 [18]	Effect of VR^d^ technology on the rehabilitation of upper and lower limb motor function in stroke patients	8	EG^e^: 164; CG^f^: 143	FMA-UE^g^	4.27	2.47 to 6.06	≤.001	0	3.033
Laver et al, 2017 [19]	To determine the efficacy of VR compared with an alternative intervention or no intervention on upper limb function and activity	10	EG: 191; CG: 172	FMA-UE	3.30	1.29 to 5.32	.001	0	4.867
Maier et al, 2019 [20]	To evaluate the efficacy of specific VR and nonspecific VR systems for rehabilitating upper limb function and activity after stroke	17	EG: 704; CG: 551	FMA-UE	0.21	0.08 to 0.33	.002	12	7.950
Zhong et al, 2019 [21]	To evaluate the VR technology on the clinical curative effect of upper limb function in patients undergoing cerebral apoplexy hemiplegia rehabilitation	17	EG: 283; CG: 274	FMA-UE	5.33	3.54 to 7.13	≤.001	22	8.117
Domínguez-Téllez et al, 2020 [2]	To evaluate game-based VR interventions to improve upper limb motor function and quality of life after stroke	9	EG: 154; CG: 148	FMA-UE	1.53	0.51 to 2.54	.003	92	3.417
Mekbib et al, 2020 [22]	To evaluate the overall effectiveness of VR therapies compared with that of conventional therapies in the recovery of upper limb functions across the 3 ICF^h^ domains	19	EG: 363; CG: 345	FMA-UE	3.84	0.93 to 6.75	.01	64	9.950

^a^k: number of primary studies.

^b^LL: lower limit.

^c^UL: upper limit.

^d^VR: virtual reality.

^e^EG: experimental group.

^f^CG: control group.

^g^FMA-UE: Fugl-Meyer Assessment Upper Extremity Scale.

^h^ICF: International Classification of Functioning, Disability and Health.

**Table 2:** Characteristics of the included studies for balance.

Author(s), year	Aim	k^a^	Sample size	Measuring instrument	Effect size			I^2^	k_adj_
					Effect size	95% CI (LL^b^ to UL^c^)	*P* value		
Gibbons et al, 2016 [23]	Effects of VR^d^ on lower limb outcomes in stroke patients	9	EG^e^: 104; CG^f^: 95	BBS^g^, FRT^h^	0.42	0.11 to 0.73	.42	10	4.077
Iruthayarajah et al, 2017 [24]	To evaluate the effectiveness of VR interventions in improving balance in a chronic stroke population	12	EG: 132; CG: 142	BBS	0.506	0.259 to 0.753	≤.001	2.622	5.012
Mohammadi et al, 2019 [25]	To evaluate the effect of VR on balance as compared with that of conventional therapy alone poststroke	13	EG: 161; CG: 153	BBS, FRT, PAS^i^	0.64	0.36 to 0.92	.083	36.7	5.530
Liang et al, 2020 [26]	To evaluate the effectiveness of VR technology in promoting balance and walking function rehabilitation in stroke	17	EG: 215; CG: 217	FRT, PAS	4.09	2.20 to 5.97	≤.001	84.5	8.513

^a^k: number of primary studies.

^b^LL: lower limit.

^c^UL: upper limit.

^d^VR: virtual reality.

^e^EG: experimental group.

^f^CG: control group.

^g^BBS: Berg Balance Scale.

^h^FRT: functional reach test.

^i^PAS: Postural Assessment Scale.

**Table 3:** Characteristics of the included studies for walking ability.

Author(s), year	Aim	k^a^	Sample size	Measuring instrument	Effect size			I^2^	k_adj_
					Effect size	95% CI (LL^b^ to UL^c^)	*P* value		
Iruthayarajah et al, 2017 [24]	To evaluate the effectiveness of VR^d^ interventions in improving balance in a chronic stroke population	13	EG^e^: 145; CG^f^: 166	TUG^g^	0.367	0.134 to 0.601	.002	0	5.467
Liang et al, 2020 [26]	To evaluate the effectiveness of VR technology in promoting balance and walking function rehabilitation in stroke	18	EG: 271; CG: 271	TUG	–2.79	–4.88 to –0.69	.009	86.2	12.267

^a^k: number of primary studies.

^b^LL: lower limit.

^c^UL: upper limit.

^d^VR: virtual reality.

^e^EG: experimental group.

^f^CG: control group.

^g^TUG: Timed Up and Go.

### Methodological Quality of Included Studies

In this study, the methodological quality of 10 meta-analysis articles was evaluated. Their scores were all >4, which was a satisfactory result. The specific scores are shown in Table 4. Moreover, the reliability of the quality evaluation was high (ICC=0.96, 95% CI 0.93-0.98, *P*<.001).

**Table 4:** Methodological quality of the included studies.

	1^a^	2^b^	3^c^	4^d^	5^e^	6^f^	7^g^	8^h^	9^i^	10^j^	11^k^	Total score
Yang et al, 2013 [18]	1	0	1	?^l^	1	1	1	?	1	1	0	8
Gibbons et al, 2016 [23]	1	1	1	1	1	1	1	?	1	0	0	8
Laver et al, 2017 [19]	1	1	1	1	0	0	1	?	?	0	0	5
Iruthayarajah et al, 2017 [24]	1	0	1	1	1	1	1	?	?	0	0	6
Mohammadi et al, 2019 [25]	1	1	1	1	1	1	1	?	1	0	1	9
Maier et al, 2019 [20]	0	1	0	0	1	1	1	?	1	1	1	6
Zhong et al, 2019 [21]	1	1	1	?	1	1	1	?	1	1	1	7
Domínguez-Téllez et al, 2020 [2]	1	1	1	1	1	1	1	?	1	0	1	9
Liang et al, 2020 [26]	1	1	1	?	1	1	1	?	1	1	0	8
Mekbib et al, 2020 [22]	1	0	1	0	0	0	1	?	1	0	1	5

^a^Whether the preliminary design scheme was provided.

^b^Whether the included studies and data extraction were repetitive.

^c^Whether an extensive and comprehensive literature search was carried out.

^d^Whether the publication was considered.

^e^Whether the list of included and excluded studies was provided.

^f^Whether the characteristics of the included studies were described

^g^Whether the scientificity of the included studies was evaluated and reported.

^h^Whether the scientificity of the included studies was appropriately used in the derivation of conclusions.

^i^Whether it was appropriate to summarize the research results.

^j^Whether the possibility of publication bias was evaluated.

^k^Whether the relevant conflicts of interest were explained.

^l^Unknown.

### Upper Limb Function

**Figure 2:**
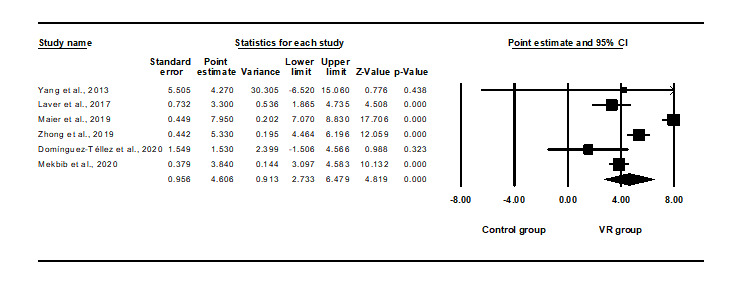
Meta-meta-analysis of the effects of virtual reality (VR) interventions on upper limb function. The bottom row describes a combined overall effect of treatment which random-effects models were used to estimate.

Evidence for VR training helping to improve stroke patients’ upper limb function was provided by 7 meta-analysis studies, and significant differences were found in these studies. After accounting for overlap, 6 studies were finally confirmed. As shown in Figure 2, the meta-meta-analysis showed that the effect of VR-based intervention on stroke had a statistically large effect size (SMD 4.606, 95% CI 2.733-6.479, *P*<.05; Figure 2), and considerable heterogeneity was found (Q=62.851, *I^2^*=92.045). The intercept of the Egger Test for publication bias was –1.44, *P*=.671.

### Balance Function

**Figure 3:**
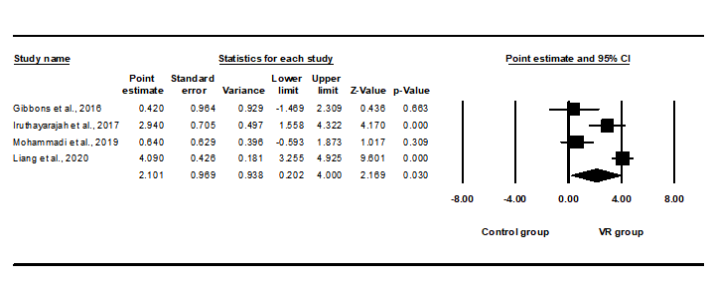
Meta-meta-analysis of the effects of virtual reality (VR) training on balance. The bottom row describes a combined overall effect of treatment which random-effects models were used to estimate.

Evidence for VR training helping to improve stroke patients’ balance ability was provided by 11 meta-analysis studies, and significant differences were found in 10 studies. After accounting for overlap, 4 studies were finally confirmed. As shown in Figure 3, the meta-meta-analysis showed that the efficacy of VR-based intervention on balance after stroke had a statistically large effect size (SMD 2.101, 95% CI 0.202-4.000, *P*<.05), and considerable heterogeneity was found (Q=27.061, *I^2^*=88.914). The intercept of the Egger Test for publication bias was –7.09, *P*=.222.

### Lower Limb Function

Evidence for VR training helping to improve lower extremity function in stroke patients was provided by 8 meta-analyses, and 8 studies showed significant differences. After accounting for overlap, only 2 studies met the condition of k_adj_ >3, and therefore, the effect size was not summarized.

## Discussion

### Principal Findings

The purpose of this meta-meta-analysis was to quantify the impact of VR training on motor performance in patients poststroke by summarizing the current mid-quality and high-quality meta-analysis results. It found that VR training showed a significant improvement in upper limb function (SMD 4.606) and balance ability (SMD 2.101), which was consistent with the results of most previous meta-analysis articles. According to the included meta-analysis studies, there were 7 studies on the effect of VR training on the upper extremities and 10 studies on balance. Our conclusions provided strong evidence for VR-induced improvement and confirmed the potential therapeutic benefits of VR training.

In the included meta-analysis studies, the FMA-UE was used to evaluate the upper extremities. It includes 33 items related to proximal and distal upper extremity movement, mainly to evaluate the reflex activity, motor control, and muscle strength of the upper extremity on the hemiplegic side. FMA-UE is widely applied as the outcome index of rehabilitation tests and to record recovery after stroke [27]. Unfortunately, due to the limited number of studies, it is impossible to test the dose effect for stroke patients with different degrees of dyskinesia to determine the most effective intervention dose of VR. The BBS was used by 3 of 4 included studies, showing a greater degree of improvement. Balance ability is a crucial step to improve the motor ability of stroke patients, although rehabilitation often involves many functions, such as visual function, vestibular function, and somatosensory receptors. However, based on the existing meta-analysis studies, VR training can effectively ameliorate these functions, leading to a greater degree of improvement in balance. It is worth noting that a customized balance rehabilitation system seems to have the same curative effect as a commercial virtual balance system, suggesting that stroke patients can carry out rehabilitation training through commercial virtual games [24]. In this meta-meta-analysis, there was a specific deviation in the effect size of these meta-analysis articles that can be considered a result of the different studies included. In addition, statistical differences were not excluded. In terms of gait, 2 meta-analysis results in this study showed moderate effects on improving walking ability. Due to the lack of sufficient qualified meta-analysis studies, no meta-meta-analysis of walking ability was performed, but its influence cannot be ignored. Due to decreased walking ability and fear of falling, stroke patients’ motor ability is weakened, which is unfavorable for recovery [28].

Optimizing and strengthening brain compensation mechanisms are essential for dyskinesia [29]. The virtual environment created by VR technology can promote the illusion of body movement, strengthen the activation of the motor brain area with a sense of immersion, mobilize changes in brain neuroplasticity, reconstruct the cell synapses of the nervous system, and realize the direct training of the central nervous system, which plays a significant role in the reorganization and recovery of neural structures after stroke [30,31]. Compared with other interventions, VR training has many potential advantages. For example, the cost of current VR equipment is relatively low. Only a screen and VR system are needed for the completion of the intervention. Second, repetitive task training has been proven to be effective in improving the motor ability of stroke patients. Current VR equipment is more convenient to carry; thus, patients can carry out regular training in their own homes. Third, current VR systems are compatible with other systems, which makes it more convenient for training. All of these cannot be realized by conventional therapy. Without increasing the number of rehabilitation therapists, these are conducive to improving the efficacy of rehabilitation and reducing patients’ medical burden [32].

VR training includes 4 components that could work together to ensure success: intensive therapy, motivating therapy through exercise games, stimulation of motor learning, and positive feedback between the stimulus and the response. Therefore, a single mechanism cannot explain the impact of VR training on stroke patients, which may be the result of both psychological mechanisms (such as self-efficacy, reward mechanism, and emotion) and physiological mechanisms (vestibular and somatosensory receptors) [33-35]. With the development of medical imaging, researchers have begun to explore the effects of VR training on the brain functions of stroke patients. Although the current research is still in its infancy, with the deepening of research, the effectiveness of VR training will soon benefit from more direct evidence [18,19,36-38].

This study conducted a meta-meta-analysis on the current meta-analysis data for VR training in improving stroke, which strengthened the evidence on this topic. After a series of controlled experiments and strict methodological quality assessment, confidence in applying VR technology in clinical rehabilitation was increased. Nevertheless, there were still some limitations in this study. First, the retrieval language was confined to English or Chinese; thus, publications in other languages may have been missed. Second, since this meta-meta-analysis was based on previously published meta-analysis articles, primary research without meta-analysis may be omitted. Finally, our primary problem was that the results showed a high degree of statistical heterogeneity. Therefore, the results should be interpreted with caution.

### Conclusion

In general, the current evidence supports that VR, which has a medium to large effect size, is beneficial for stroke patients’ motor ability, especially in the upper extremities and for balance. However, no specific rehabilitation treatment has been formulated to date, likely due to different motor ability levels. Consequently, future research on this topic requires randomized controlled trials with larger sample sizes and longer duration to verify that VR training is the best treatment for improving stroke patients’ motor performance and to verify the optimal type, frequency, duration, and cycle of VR training for patients with different motor abilities.

## Appendix

Multimedia Appendix 1 Search strategy.

## Abbreviations

BBS: Berg Balance Scale
CMA: Comprehensive Meta-Analysis
FMA-UE: Fugl-Meyer Assessment Upper Extremity Scale
ICC: intraclass correlation coefficient
SMD: standardized mean difference
VR: virtual reality
