# Supplementary material for: Effects of Virtual Reality Training on Upper Limb Function and Balance in Stroke Patients: Systematic Review and Meta-Meta-Analysis
Source: J Med Internet Res. 2021 Oct 12;23(10):e31051. doi: 10.2196/31051 (PMC8548971; doi:10.2196/31051)
Supplement: Multimedia Appendix 1 [file jmir_v23i10e31051_app1.docx]

Pubmed search strategy

| 1. Pubmed | |
| --- | --- |
| #1 | Search (meta[Title/Abstract]) OR (meta-analysis[Title/Abstract]) |
| #2 | Search (stroke[Title/Abstract]) OR (poststroke[Title/Abstract]) |
| #3 | Search ((((("virtual reality"[Title/Abstract]) OR ("virtual game"[Title/Abstract])) OR ("virtual video"[Title/Abstract])) OR ("Nintendo Wii, Kinect"[Title/Abstract])) OR (Xbox[Title/Abstract])) OR (exergame[Title/Abstract]) |
| #4 | #1 AND #2 AND #3 |
| #5 | Filters: Publication date to 2020/09/25.  Items found: 56 |
